# Interferon-γ Possesses Anti-Microbial and Immunomodulatory Activity on a *Chlamydia trachomatis* Infection Model of Primary Human Synovial Fibroblasts

**DOI:** 10.3390/microorganisms8020235

**Published:** 2020-02-10

**Authors:** Marisa Di Pietro, Simone Filardo, Federica Frasca, Carolina Scagnolari, Martina Manera, Vincenzo Sessa, Guido Antonelli, Rosa Sessa

**Affiliations:** 1 Section of Microbiology, Department of Public Health and Infectious Diseases, Sapienza University, 00185 Rome, Italy; marisa.dipietro@uniroma1.it (M.D.P.); martina.manera@uniroma1.it (M.M.); rosa.sessa@uniroma1.it (R.S.); 2 Laboratory of Virology, Department of Molecular Medicine, affiliated to Istituto Pasteur Italia–Cenci Bolognetti Foundation, Sapienza University, 00185 Rome, Italy; federica.frasca@uniroma1.it (F.F.); carolina.scagnolari@uniroma1.it (C.S.); guido.antonelli@uniroma1.it (G.A.); 3 Department of Orthopedics, San Giovanni Calibita-Fatebenefratelli Hospital, 00186 Rome, Italy; v.sessa@libero.it; 4 Microbiology and Virology Unit, Hospital “Policlinico Umberto I”, Sapienza University, 00185 Rome, Italy

**Keywords:** *Chlamydia trachomatis*, human synovial fibroblasts, innate immunity, interferon-α, interferon-β, interferon-γ

## Abstract

*Chlamydia trachomatis*, an obligate intracellular pathogen, is the most common cause of bacterial sexually transmitted diseases, and it is potentially responsible for severe chronic sequelae, such as reactive arthritis. To date, details of the mechanisms by which Chlamydiae induce innate antimicrobial pathways in synovial fibroblasts, are not well characterized; therefore, herein, we investigated the effects of interferon (IFN)α, IFNβ, and IFNγ on the infection, and replication phases of the *C. trachomatis* developmental cycle, as well as on the induction of pattern recognition receptors (PRRs) and IFN-related pathways. To do so, we set up an in vitro chlamydial-infection model of primary human synovial cells treated with IFNs before or after the infection. We then determined the number of chlamydial inclusion forming units and inclusion size, as well as the expression of toll like receptor (TLR)2, TLR3, TLR4, cyclic GMP-AMP synthase (cGAS), stimulator of IFN gene (STING), IRF9, ISG56, and GBP1. The main result of our study is the significant inhibition of *C. trachomatis* infection and replication in human synovial cells following the treatment with IFNγ, whereas IFN-I proved to be ineffective. Furthermore, IFNγ greatly upregulated all the PRRs and ISGs examined. In conclusion, IFNγ exhibited a potent anti-Chlamydia activity in human synovial cells as well as the ability to induce a strong increase of innate immune pathways.

## 1. Introduction

*Chlamydia trachomatis* is an obligate intracellular bacterium with a unique developmental cycle characterized by the extracellular infectious elementary body (EB), which invades the host cell, and the intracellular replicative reticulate body (RB), responsible for the multiplication within the host [1]. *C. trachomatis* is still an important public health problem worldwide, because of the impact of asymptomatic genital infections in both women (90%) and men (50%), favoring the onset of severe chronic complications, including reactive arthritis (ReA) in both genders [2,3,4]. It is estimated, indeed, that approximately 4–8% of patients will develop ReA one to six weeks after a urogenital *C. trachomatis* infection [5]. In 30% of all cases, ReA persists for years, compromising joint function and leading, eventually, to joint deformities and ankylosis [6,7]. The involvement of *C. trachomatis* in ReA has been supported by studies showing chlamydial DNA, RNA, and proteins in synovial fluid, as well as by in vitro studies, demonstrating the ability of *C. trachomatis* to infect synovial fibroblasts [8,9,10].

As for most bacterial infections, the host immune response, mediated by the production of a plethora of cytokines and other immune mediators, is involved in the protection against *C. trachomatis* infection, as well as the induction of an increased inflammatory state, contributing to tissue damage [11,12,13].

Generally, the first line of defense against *C. trachomatis* is the innate immune response and one of the major players is the secretion, from infected cells, of type I interferons (IFN-I) [14]. In particular, germ line encoded pattern recognition receptors (PRRs), such as toll like receptors (TLRs), and the cyclic GMP-AMP synthase-stimulator of the IFN gene (cGAS-STING) pathway, bind bacterial components, promote signaling events, and coordinate the activation of transcription factors that induce the expression of antimicrobial molecules, chemokines, and several cytokines, such as IFN-I themselves (i.e., IFNα and IFNβ) [12,14,15]. Type I IFNs signal in an autocrine and paracrine fashion through a heterodimeric transmembrane receptor (IFNAR1/IFNAR2) and, as a result, JAK-STAT pathway is activated, and several IFN stimulated genes (ISGs) are induced, promoting the recruitment of T-lymphocytes and other inflammatory cells [12,16]. Then, the T-cell dependent adaptive immune response leads to the synthesis of IFNγ (type II IFN), which seems to be the predominant response for the clearance of *C. trachomatis*. Indeed, in vivo studies on murine models of genital *C. trachomatis* infections have observed enhanced chlamydial levels in either mice with IFNγ or IFNγ receptor genes knocked-out, or in mice treated with anti-IFNγ antibodies, as compared to controls [17,18,19,20].

The host immune response against *C. trachomatis* has been widely studied in respect to chlamydial genital infection, whereas its role in chronic complications, like ReA, still needs to be investigated [12,21]. Therefore, a better understanding of how this pathogen modulated the type I and type II IFN response may provide important evidence on the pathogenesis of ReA as well as new clues for the treatment of chronic *C. trachomatis* infection. In this regard, we hypothesized that the IFNγ-mediated response might be the most effective for the clearance of *C. trachomatis*, as compared to a type I IFN mediated response.

To test our hypothesis, an in vitro infection model of primary human synovial cells was used to study the effects of IFNα, IFNβ, and IFNγ on the infection and replication phases of the *C. trachomatis* developmental cycle as well as on the induction of PRRs and IFN-related pathways. Our approach revealed an exciting mechanism by which IFNγ exhibited protective activity towards *C. trachomatis* in primary synovial cells, an effect that was mediated by a strong induction of innate immune pathways.

## 2. Materials and Methods

### 2.1. Reagents

The following IFNs were used in the present study: IFN-α, also known as natural (n)IFN-α, which is mixture of IFNα subtypes (Alfaferone, ALFA WASSERMANN, Milan, Italy); IFN-β-1a (AVONEX, Biogen, Inc., Cambridge, MA, USA); IFN-γ (Gamma Interferon, Boehringer Ingelheim, Ingelheim am Rhein, Germany).

### 2.2. Cell Culture and Culture Conditions

Primary human fibroblast-like synoviocytes (HFLS, 408K-05a, Cell Applications Inc., San Diego, CA, USA) were seeded in 80 cm2 cell culture flasks and grown in Dulbecco’s Modified Eagle Medium (DMEM) supplemented with 15% FBS, penicillin (100 U/mL) and streptomycin (100 µg/mL) at 37 °C in a humidified atmosphere with 5% CO_2_. Upon confluency (>85%), cells were passaged with brief trypsinization, and all experiments were performed using cells that were passaged at least 4 or 5 times.

McCoy cell line (ECACC, Public Health England, catalogue number 90010305, Porton Down, Salisbury, UK) was cultured in Dulbecco’s Modified Eagle Medium (DMEM, Gibco™, Gaithersburg, MD, USA) supplemented with 10% (*v*/*v*) fetal calf serum (FCS), at 37 °C in humidified atmosphere, with 5% CO_2_.

### 2.3. Propagation and Titration of C. trachomatis

*C. trachomatis* serovar D strain UW3 (VR-855, ATCC, Manassas, VA, USA) was propagated in McCoy cells, as previously described [22]. Briefly, confluent McCoy cell monolayers, grown on 25 cm^2^ cell culture flasks, were infected with chlamydial EBs by centrifugation at 754× *g* for 30 min, and then harvested by scraping after 36 to 40 h post infection. The resulting suspension was vortexed with sterile glass beads for 1 min and, after removal of cell debris by centrifugation at 250× *g* for 10 min, the supernatant, containing chlamydial EBs, was added to equal volume of 4× Sucrose Phosphate (4SP) buffer, and stored at −80 °C.

For *C. trachomatis* titration, McCoy cell monolayers, grown on 24 wells cell culture trays, were infected with 10-fold serial dilutions of bacterial stock, incubated for 48 h at 37 °C, fixed with methanol and stained with isothiocyanate-conjugated monoclonal antibody anti-C. trachomatis LPS (Merifluor^®^ Chlamydia, Meridian Bioscience Inc., Cincinnati, OH, USA), as previously described [23]. The total number of *C. trachomatis* Inclusion Forming Units (IFUs) was enumerated by counting all microscope fields using a fluorescence microscope (400× magnification).

### 2.4. Cytotoxicity of Interferons on Human Synovial Fibroblasts

Confluent human synovial fibroblast monolayers, grown on 96 wells cell culture trays, were incubated with increasing concentrations of IFNs (10, 10^2^, and 10^3^ International Units (IU)/mL) in DMEM supplemented with 15% FBS at 37 °C in humidified atmosphere with 5% CO2. After 24 h, the number of viable cells was assessed by MTT (3-(4,5-dimethylthiazol-2-yl)-2,5-diphenyltetrazolium bromide, a tetrazole) assay, as previously described [24,25].

### 2.5. Pre-Infection Treatment of Human Synovial Fibroblasts with Interferons

Confluent human synovial fibroblast monolayers, grown on glass coverslips in 24 wells cell culture trays, were pre-incubated with IFN-α, IFN-β, and IFN-γ at non-cytotoxic concentrations in DMEM supplemented with 15% FBS, at 37 °C in humidified atmosphere with 5% CO2. After 24 h, cell monolayers were washed with PBS, infected with *C. trachomatis* (MOI 10) by centrifugation at 754× *g* for 30 min and incubated at 37 °C for 28 h. Cell monolayers were then fixed with methanol and stained as described above. The total number of *C. trachomatis* IFUs was enumerated by counting all microscope fields using a fluorescence microscope (400× magnification).

### 2.6. Post-Infection Treatment of Human Synovial Fibroblast with Interferons

Confluent human synovial fibroblast monolayers, grown on glass coverslips in 24 wells cell culture trays, were infected with *C. trachomatis* (MOI 10) by centrifugation at 754× *g* for 30 min and incubated at 37 °C in humidified atmosphere with 5% CO_2_. After 3 h post infection, cell monolayers were treated with IFN-α, IFN-β, and IFN-γ at non-cytotoxic concentrations in DMEM supplemented with 15% FBS and incubated for 24 h at 37 °C. Cell monolayers were then fixed with methanol and stained as above described. The total number of *C. trachomatis* IFUs was enumerated by counting all microscope fields using a fluorescence microscope (400× magnification).

Chlamydial inclusion size, expressed as µm^2^, was also measured on an average of 100 inclusions from 50 microscope fields, for each condition from the fluorescence micrographs, by using ImageJ software (version 1.52a, NIH, Bethesda, MD, USA). The fluorescence microscope used to assess the size of chlamydial inclusion was a Brightfield transmitted light optical microscope, and the pictures were taken at the focal plane where the projected area of chlamydial inclusions was at its maximum.

### 2.7. TaqMan-based Real-Time RT-PCR Assay for PRRs and ISGs mRNA Expression

Quantitative real-time PCR for PRRs (TLR2-4, cGAS, and STING) and ISGs (IRF-9, ISG56 and interferon-induced guanylate-binding protein GBP1) was carried out with the LightCycler 480 instrument (Roche, Basel, Switzerland). Briefly, total RNA was extracted from synovial cells treated with IFN-I (IFNα or IFNβ), or IFNγ at non-cytotoxic concentrations, before or after *C. trachomatis* infection, using the RNeasy Plus Universal Tissue Mini Kit (Invitrogen, Carlsbad, CA, USA) and reverse transcribed using the High Capacity cDNA Reverse Transcription Kit (Applied Biosystems, Woburn, MA, USA), according to the manufacturer’s instruction. Primers and probes for each gene were added to the Probes Master Mix (Roche, Basel, Switzerland) at 500 and 250 nM, respectively, in a final volume of 20 μL. The housekeeping gene β-glucuronidase (GUS) was used as an internal control. GUS was selected as a good candidate for the housekeeping gene in our experimental setting, because it was constantly expressed in synovial cells after *C. trachomatis* or IFN stimulation. Gene expression values were calculated by the comparative 2^−Δ*C*t^ and 2^−ΔΔ*C*t^ methods. The mRNA levels of PRRs and ISGs were expressed as fold change calculated by setting the untreated cells as one (2^−ΔΔ*C*t^ method). The primers and probe were assayed on demand and were purchased from Integrated DNA Technologies (IDT), Clear Creek, IA, USA. The list of primers and probes is as follows: TLR2 (Hs.PT.58.21312907), TLR3 (Hs.PT.58.25887499.g), TLR4 (Hs.PT.58.38700156.g), cGAS (Hs.PT.58.20682405), STING (Hs.PT.58.20781952), IRF-9 (Hs.PT.58.3264634), and GBP1 (Hs.PT.58.27370056). The primers and probe sequences used for ISG56 were the following: Forward 5′-TGAAGAAGCTCTAGCCAACATGTC-3′; Reverse 5′-GAGCTTTATCCACAGAGCCTTTTC-3′; Probe [6FAM]TATGTCTTTCGATATGCAGCCAAGTTTTACCG[TAM].

### 2.8. Statistical Analysis

All values were expressed as mean ± standard deviation (SD) of three replicates from three independent experiments. Comparison of means was performed by using a two-tailed *t*-test for independent samples. A value of *p* < 0.05 was considered statistically significant.

## 3. Results

### 3.1. Cytotoxicity of IFN-I/II on Human Synovial Fibroblasts

We investigated the cytotoxicity of type I and II IFNs on human synovial fibroblasts via MTT assay. IFNα, IFNβ, and IFNγ did not show any toxic effects on cell viability up to 10^3^ IU/mL (Figure S1). Hence, the concentrations 10^2^ and 10^3^ IU/mL were chosen for the subsequent experiments.

### 3.2. Effects of IFN-I/II on C. trachomatis Infection

We evaluated the effects of IFNα, IFNβ, and IFNγ on the infection and replication phases of *C. trachomatis* developmental cycle in an in vitro infection model of primary human synovial fibroblasts. In this regard, a high MOI (MOI = 10) of *C. trachomatis* was chosen, since human primary synovial fibroblasts possessed a very low susceptibility to the infection.

**Figure 1:**
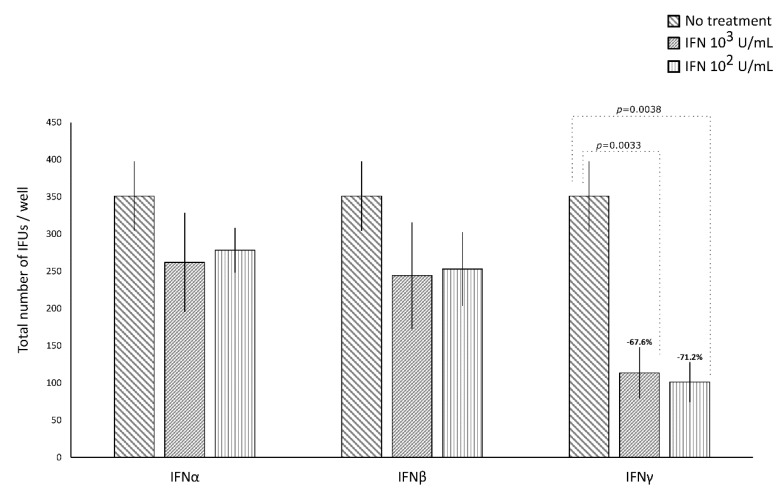
Anti-chlamydial activity of interferon (IFN)α, IFNβ, and IFNγ following the pre-infection treatment of human synovial fibroblasts. Confluent monolayers pre-treated with the IFNs (10^2^ or 10^3^ IU/mL) for 24 h, were infected with *C. trachomatis*. The total number of IFUs was determined by fluorescence microscopy, counting all green-stained inclusions at 400× magnification.

**Figure 2:**
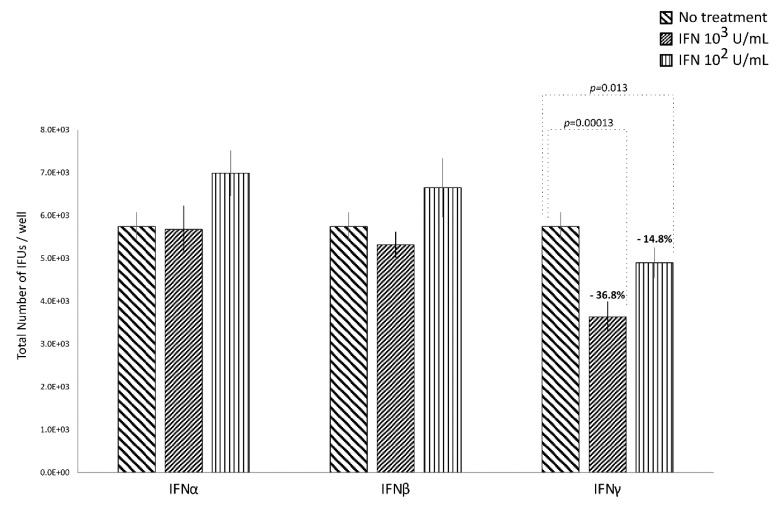
Anti-chlamydial activity of IFNα, IFNβ, and IFNγ following the post-infection treatment of human synovial fibroblasts. Confluent monolayers were infected with *C. trachomatis* and, then, treated with the IFNs (10^2^ or 10^3^ IU/mL) for 24 h. The total number of Inclusion Forming Units (IFUs) was determined by fluorescence microscopy, counting all green-stained inclusions at 400× magnification.

IFNα and IFNβ (10^2^ and 10^3^ IU/mL), in either the pre-infection or the post-infection treatments of *C. trachomatis*-infected human synovial fibroblasts, did not show any significant decrease in the number of chlamydial IFUs, as compared to uninfected cells (Figure 1 and Figure 2).

By contrasts, IFNγ, as shown in Figure 1, did possess anti-chlamydial activity in either the pre-infection or the post-infection treatment of *C. trachomatis* infected human synovial fibroblasts. In particular, a statistically significant decrease in the number of chlamydial IFUs was observed in cells pre-treated with IFNγ, at either 10^2^ or 10^3^ IU/mL, for 24h and then infected by *C. trachomatis*, as compared to uninfected cells (*p* < 0.01). A similar trend was also observed in synovial fibroblasts infected by *C. trachomatis* and then treated with IFNγ for 24 h, as evidenced by Figure 2 (*p* < 0.05 and *p* < 0.001 at 10^2^ and 10^3^ IU/mL, respectively).

Furthermore, a higher reduction rate in the number of chlamydial IFUs was observed in the IFNγ pre-infection treatment (67.6% at 10^3^ IU/mL and 71.2% at 10^2^ IU/mL) rather than in the IFN-γ post-infection treatment (36.8% at 10^3^ IU/mL and 14.8% at 10^2^ IU/mL, *p* < 0.01).

Lastly, in either the pre-infection or the post-infection treatment of human synovial fibroblasts, the anti-chlamydial activity of IFNγ was not dose-dependent.

**Figure 3:**
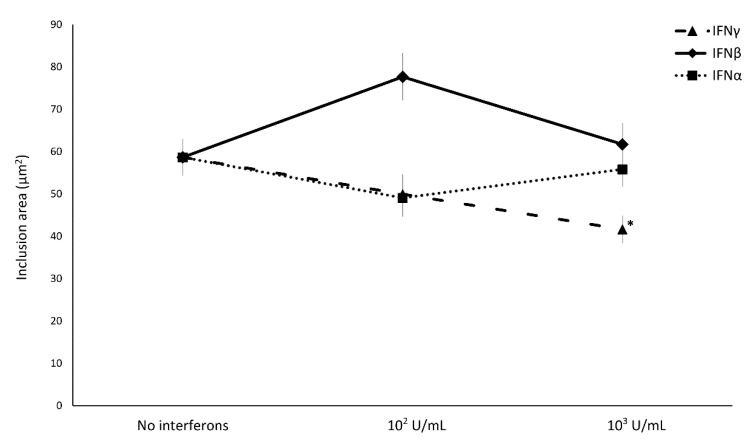
Effect of IFNα, IFNβ, and IFNγ on *C. trachomatis* inclusion size in human synovial fibroblasts. Confluent monolayers were infected with *C. trachomatis* and, then, treated with the IFNs (10^2^ or 10^3^ IU/mL) for 24 h. The chlamydial inclusion area, expressed as µm^2^, was measured by fluorescence microscopy at 400× magnification. * *p* < 0.01 vs. untreated cells.

Concerning the effect of IFN-I/II on *C. trachomatis* intracellular replication, IFNα and IFNβ did not induce any decrease in the size of chlamydial inclusions, whereas only the treatment with IFNγ showed a reduction in chlamydial inclusion size with a direct correlation to the concentration used (Figure 3, *p* < 0.01).

### 3.3. Immune response of human synovial fibroblasts to C. trachomatis infection

Human synovial cells infected by *C. trachomatis* showed a statistically significant upregulation of TLR2 and TLR3 as compared to uninfected cells, with the highest fold change observed for TLR2 (32-fold, *p* < 0.05) followed by TLR3 (4-fold, *p* < 0.05). IRF9, ISG56, and GBP1, correlated to the downstream IFN signaling pathways, were also upregulated in chlamydia-infected synovial cells, with the highest fold change for ISG56 (26-fold, *p* < 0.05), followed by GBP1 (7-fold, *p* < 0.05), and IRF9 (2.4-fold, *p* < 0.05). By contrast, TLR4, cGAS and STING expression levels were not significantly increased in *C. trachomatis*-infected synovial cells as compared to uninfected cells (Table 1).

### 3.4. Effects of IFN-I/II on PRRs and IFN-related Signaling Pathways

Having observed that IFNγ exhibited a more efficient inhibition of chlamydial growth in synovial fibroblasts as compared to IFNα/β at the concentration of 10^3^ IU/mL, we next evaluated whether the addition of IFNα, IFNβ, or IFNγ in synovial cells, before or after *C. trachomatis* infection, caused a different expression of PRRs (TLRs, cGAS, and STING) and ISGs (IRF-9, ISG56, and GBP1).

**Figure 4:**
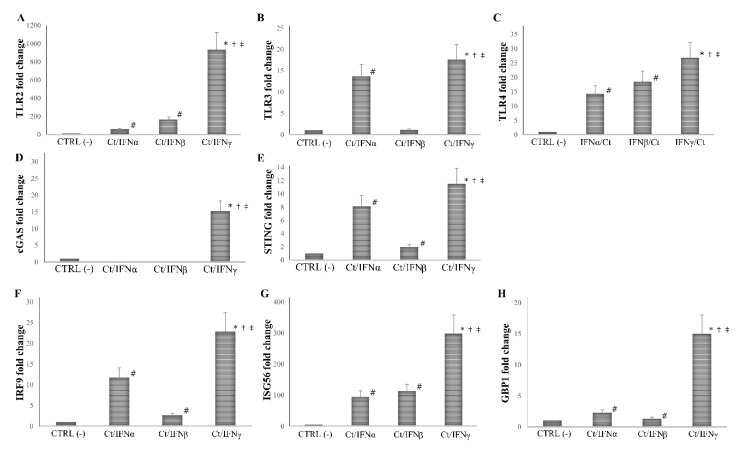
Expression of innate immune response-related genes following the pre-infection treatment of *C. trachomatis* in human synovial fibroblasts. Synovial cells were treated with IFNα, IFNβ, or IFNγ (10^3^ IU/mL) for 24 h and, then, infected with *C. trachomatis*. Data are expressed as fold change value (2^−ΔΔCt^ method) in mRNA levels compared to baseline values observed in untreated and uninfected synovial cells [set to 1 and indicated in the graphs as CTRL (-)]. Expression of (**A**) TLR2, (**B**) TLR3, (**C**) TLR4, (**D**) cGAS, (**E**) STING, (**F**) IRF9, (**G**) ISG56, and (**H**) GBP1. * *p* < 0.01 vs. CTRL (-); # *p* < 0.05 vs. CTRL (-); † *p* < 0.05 vs. Ct/IFNα; ‡ *p* < 0.05 vs. Ct/IFNβ.

We found that the pre-treatment with IFNγ induced a more than 10-fold increase in the expression of all PRRs and ISGs analyzed, as compared to the untreated cell control (*p* < 0.01 for all genes), and the highest level of mRNA detection following IFNγ pre-treatment was observed for TLR2 (1000-fold), and ISG56 (300-fold). As expected, the pre-treatment with IFNα/β caused an induction of at least 2-fold of most PRRs and ISGs studied (*p* < 0.05), with the only exception of cGAS, which was not induced after IFN-I pre-treatment (*p* > 0.05). Of note, both IFNα/β induced a lower expression of TLR2, TLR3, TLR4, cGAS, STING, IRF9, ISG56, and GBP1 as compared to IFNγ (*p* < 0.05, Figure 4).

**Figure 5:**
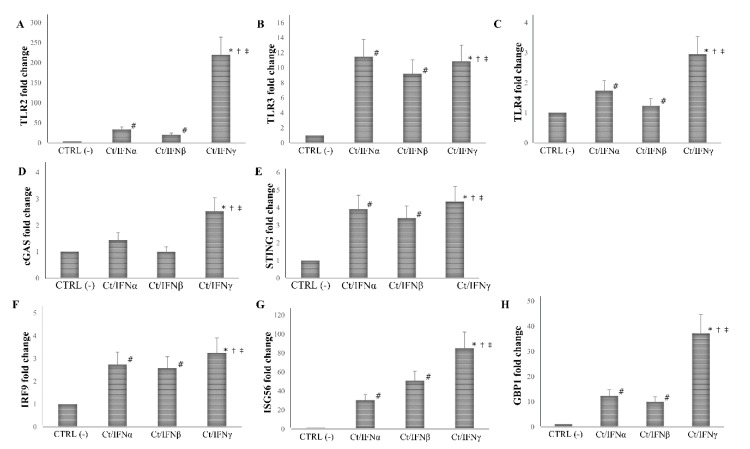
Expression of innate immune response-related genes following the post-infection treatment of *C. trachomatis* in human synovial fibroblasts. Synovial cells were infected with *C. trachomatis* and, then, treated with IFNα, IFNβ, or IFNγ (10^3^ IU/mL) for 24 h. Data are expressed as fold change value (2^−ΔΔ*C*t^ method) in mRNA levels compared to baseline values observed in untreated and uninfected synovial cells [set to 1 and indicated in the graphs as CTRL (-)]. Expression of (**A**) TLR2, (**B**) TLR3, (**C**) TLR4, (**D**) cGAS, (**E**) STING, (**F**) IRF9, (**G**) ISG56, and (**H**) GBP1. * *p* < 0.01 vs. CTRL (-); # *p* < 0.05 vs. CTRL (-); † *p* < 0.05 vs. Ct/IFNα; ‡ *p* < 0.05 vs. Ct/IFNβ.

A similar trend was observed when IFN-I/II were added after *C. trachomatis* infection. Indeed, the post-treatment with IFNγ induced higher levels of TLR2, TLR4, cGAS, ISG56, and GBP1 as compared to IFNα/β (*p* < 0.05), except for TLR3, STING, and IRF-9, whose expression was not statistically different (Figure 5). Moreover, the ability of either IFN-I or IFN-II to induce the PRRs and ISGs examined was significantly lower in the IFNs post-infection treatment, as compared to the IFNs pre-infection treatment of human synovial fibroblasts.

## 4. Discussion

*C. trachomatis*, an obligate intracellular pathogen, is the most common cause of bacterial sexually transmitted diseases worldwide, and it is potentially responsible for severe chronic sequelae, such as ReA [2,26].

The host innate immunity represents the first line defense against *C. trachomatis* infection and, amongst all the inflammatory cytokines involved, type I and II IFNs, originally believed to exclusively participate in antiviral responses, have recently acquired importance for their protective role in the anti-bacterial defense [12,14,27].

Herein, we investigated, for the first time, the effects of type I and II IFNs in an in vitro infection model of *C. trachomatis* on primary human synovial fibroblasts. The main result of our study is the significant inhibition of *C. trachomatis* infection and intracellular replication in human synovial cells following the treatment with IFNγ, whereas IFN-I proved to be ineffective.

The protective effect of IFNγ is consistent with the fact that the treatment of *C. trachomatis*-infected human synovial fibroblasts with this cytokine appeared to greatly upregulate all the innate immune genes examined (TLR2, TLR3, TLR4, cGAS, STING, IRF9, ISG56, and GBP1), with the highest increase observed for TLR2 and ISG56. To further confirm the importance of IFNγ in the host immune defense towards *C. trachomatis*, the response of untreated synovial cells to chlamydial infection appeared to be mediated only by the increased expression of pattern recognition receptors TLR2 and TLR3, followed by the activation of IRF9 and the expression of ISG56 and GBP1, whereas TLR4 and the pathway cGAS/STING were not engaged.

It is well known that PRR-dependent signaling-pathways contribute to the local immune response towards invading pathogens, including *C. trachomatis*, via the induction of inflammatory mediators and IFNs [28,29,30]. Particularly, TLR2 and TLR4 have been widely related to *C. trachomatis* infection in different cell types and, recently, cGAS, a cytosolic DNA sensing PRR, has been shown to detect *C. trachomatis* nucleic acids [15,29]. The activation of TLR3 following a chlamydial infection has also been recently demonstrated in human Sertoli cells, an epithelial cell line of the testis, suggesting that this immune mediator could also be involved in the recognition of *C. trachomatis* [31]. In addition, data from in vivo infection models demonstrated that TLR2 knockout mice had a more severe disease, as well as an intense and prolonged chlamydial infection, as compared to wild type mice, and TLR3 and STING played a key role in the activation of a multitude of inflammatory modulators [32,33,34].

Following the recognition of *C. trachomatis* by the respective PRR, different downstream signaling pathways are activated (i.e., STING), leading to the transcription of IFN-I [12]. Once these cytokines are produced and released, they can exert their effects via the dimerization of STAT1/2 with IRF9 to form a complex that, in turn, translocates to the nucleus, where it binds interferon-stimulated response elements (ISREs), leading, then, to the expression of ISGs, including ISG56 and GBP1, that possess anti-chlamydial activities [15,35,36].

In this study, *C. trachomatis* was able to activate a variety of innate immune pathways in synovial cells, like TLR2, TLR3, and ISGs (ISG56 and GBP1), whereas TLR4, cGAS, and STING were only marginally increased, suggesting a lack of involvement of the latter in the synovial innate immune response to *C. trachomatis* infection. This is, in fact, in agreement with in vitro studies showing that, in epithelial cells, non-TLR4 ligands are involved in the inflammatory response to *C. trachomatis* [37]. Furthermore, it has been demonstrated, in an in vivo model of the *Chlamydia muridarum* infection, the lack of involvement of CD14 (an accessory protein essential for TLR4 recognition) in the immunopathogenesis of the infection, suggesting that TLR4 might not be important for the initial signaling pathway for pro-inflammatory cytokine production during chlamydial infection [38].

Despite the activation of the host immune pathways, the synovial cell response to *C. trachomatis*, observed in our study, was not effective against the infection, suggesting the possibility that this pathogen has evolved ways of inhibiting these molecular sensors in synovial fibroblasts, potentially contributing to the development of ReA. In fact, it has been demonstrated that *C. trachomatis* inhibited the downstream signaling pathways of TLR4 [39], as well as the important role of STING in chlamydial growth [15], further strengthening our hypothesis.

More interestingly, the observation, in our study, that the treatment of synovial cells with IFNγ was highly effective in inhibiting *C. trachomatis* infection and growth as well as inducing TLR2 and ISG56-related gene expression, suggests the importance of IFNγ-mediated immune response in the clearance of *C. trachomatis* infection. To date, IFNγ activity as an anti-chlamydial agent is believed to be mostly mediated by its ability to reduce tryptophan availability via the upregulation of the enzyme indoleamine 2,3-dioxygenase (IDO)-1, which metabolizes tryptophan into kynurenine, inhibiting chlamydial replication [40]. Nevertheless, in our study, the anti-chlamydial effect of IFNγ were present even after replenishing the intracellular and extracellular tryptophan pool by adding fresh culture media after the *C. trachomatis* infection. Therefore, the increased expression of TLR2 and ISGs (ISG56 and GBP1) in IFNγ-treated human primary synovial fibroblasts, hints to possible other pathways involved in the protective role of this cytokine towards *C. trachomatis*. For example, the induction of TLR2 by IFNγ is well known to promote the activation of antimicrobial defense systems (i.e., innate and adaptive immune responses, through NFKB and IRF3) [41], thus potentially hindering chlamydial growth. In addition, ISG56 has been proposed to regulate an important balance between inflammatory and IFN gene programs, facilitating an optimal host response to microbial challenge, as it might happen against *C. trachomatis* [42]. Lastly, Tietzel et al. [43] showed that the downregulation of GBP1 lead to a decrease in the inhibitory effect of IFNγ towards *C. trachomatis* replication.

In addition, IFNγ possessed a much higher anti-chlamydial activity and induced a stronger increase in the expression levels of the PRRs and ISGs examined when added before, rather than after, *C. trachomatis* infection. This is not surprising, since IFNγ, as a part of the host adaptive immune system [44], might render the cell less susceptible to *C. trachomatis* infection via the fine regulation of the complex network of immune modulators, and this deserves further investigation.

It is particularly intriguing that, in our study, IFNα and IFNβ were ineffective towards chlamydial infection and replication in human synovial fibroblasts, since the protective role of IFN-I in the host defense to *C. trachomatis* has been evidenced in cervical epithelial cells [45]. This difference, in fact, highlights the possibility that cell-type specific mechanisms might be stimulated by IFNs during *C. trachomatis* infection.

It is also known that the antimicrobial effects of IFNs are mediated by their receptors on the cell surface. Types I and II IFNs bind distinct cell surface receptor complexes: the IFNAR1/R2 (IFN-α/β receptor, R) and the IFNGR1/R2 (IFN-γR), respectively [46,47]. Although the IFNγR and IFN-α/βR are ubiquitously expressed on virtually all nucleated cells [48], a general mechanism for cells to control IFN activity is by varying their receptor concentration [49,50]. However, a plethora of additional factors might explain the different anti-Chlamydia effects of type I and II IFNs, such as, for example, IFN-binding affinity toward the receptor subunits, the duration of binding, the induction of feedbacks on the receptors, on their activation and signaling [49,50], underlining the increasing complexity of this regulatory network.

IFN-I is also able to inhibit IFNγ production and its activity [51], suggesting that the complex cross talk between immune cell activity and IFNγ might promote pro-inflammatory responses, that, in turn, can be detrimental for the host tissues, contributing to the etiopathogenesis of diseases, such as ReA.

It is important to notice that the levels of interferons in women with a genital *C. trachomatis* infection are usually lower than 0.02 U/mL, as evidenced by several studies in the literature [11,52,53], much lower than the IFN concentrations used in our study, suggesting that the strong anti-chlamydial activity of IFNγ might also have relevant clinical implications.

The strength of our study lies in the adoption of an in vitro *C. trachomatis* infection model using primary human synovial fibroblasts, that might better mirror the physiology of the human organism, although, at the same time, the utilization of a monoculture might represent a limitation, since it does not allow to investigate the contribution of the inflammatory cells recruited to the site of infection.

In conclusion, IFNγ exhibits not only a potent anti-chlamydia activity in synovial cells, but also the ability to induce a strong increase of innate immune pathways. In the future, it will be interesting to investigate the expression of mRNA and protein levels of host immune mediators, including IDO and IFN receptors during the entire developmental cycle of *C. trachomatis* in human synovial fibroblasts following IFNs treatment, in order to better define the fine regulation of the host cell innate immunity.

## Figures and Tables

**Table 1:** Expression of pattern recognition receptors (PRRs) and IFN stimulated genes (ISGs) in human synovial fibroblasts after *C. trachomatis* infection.

	Synovial Fibroblasts (A)	Synovial Fibroblasts Infected with *C. trachomatis* (B)	Fold Change (C)
TLR2	0.070 ± 0.01	2.243 ± 0.44	31.975 *
TLR3	2.091 ± 0.41	8.267 ± 1.65	3.952 *
TLR4	3.189 ± 0.63	5.236 ± 1.04	1.641
cGAS	0.777 ± 0.15	0.645 ± 0.12	0.830
STING	5.065 ± 1.01	8.710 ± 1.74	1.720
IRF9	5.736 ± 1.14	13.737 ± 2.74	2.394 *
ISG56	0.730 ± 0.14	18.912 ± 3.78	25.880 *
GBP1	3.054 ± 0.61	21.406 ± 4.28	7.007 *

Expression of PRRs (TLR2, TLR3, TLR4, cGAS, and STING) and ISGs (IRF9, ISG56 and GBP1) were calculated using 2^−ΔCt^ (A and B) or 2^−ΔΔ*C*t^ methods (C); * *p* < 0.05 vs. uninfected cells.

## Supplementary Materials

The following are available online at https://www.mdpi.com/2076-2607/8/2/235/s1, Figure S1: Cytotoxic activity of IFNα, IFNβ and IFNγ on human primary synovial fibroblast.

## References

[1] Abdelrahman Y.M., Belland R.J. (2005). The Chlamydial developmental cycle. FEMS Microbiol. Rev..

[2] GBD 2016 Disease and Injury Incidence and Prevalence Collaborators (2017). Global, regional, and national incidence, prevalence, and years lived with disability for 328 diseases and injuries for 195 countries, 1990–2016: A systematic analysis for the Global Burden of Disease Study 2016. Lancet.

[3] Di Pietro M., Schiavoni G., Sessa V., Pallotta F., Costanzo G., Sessa R. (2013). *Chlamydia pneumoniae* and osteoporosis-associated bone loss: A new risk factor?. Osteoporos. Int..

[4] Mylonas I. (2012). Female genital *Chlamydia trachomatis* infection: Where are we heading?. Arch. Ginecol. Obstet..

[5] Denison H.J., Curtis E.M., Clynes M.A., Bromhead C., Dennison E.M., Grainger R. (2016). The incidence of sexually acquired reactive arthritis: A systematic literature review. Clin. Rheumatol..

[6] Di Pietro M., Filardo S., Romano S., Sessa R. (2019). *Chlamydia trachomatis* and *Chlamydia pneumoniae* Interaction with the Host: Latest Advances and Future Prospective. Microorganisms.

[7] Zeidler H., Hudson A.P. (2016). Coinfection of Chlamydiae and other Bacteria in Reactive Arthritis and Spondyloarthritis: Need for Future Research. Microorganisms.

[8] Kumar P., Bhakuni D.S., Rastogi S. (2014). Diagnosis of *Chlamydia trachomatis* in patients with reactive arthritis and undifferentiated spondyloarthropathy. J. Infect. Dev. Ctries..

[9] Gérard H.C., Carter J.D., Hudson A.P. (2013). *Chlamydia trachomatis* is present and metabolically active during the remitting phase in synovial tissues from patients with chronic Chlamydia-induced reactive arthritis. Am. J. Med. Sci..

[10] Hanada H., Ikeda-Dantsuji Y., Naito M., Nagayama A. (2003). Infection of human fibroblast-like synovial cells with *Chlamydia trachomatis* results in persistent infection and interleukin-6 production. Microb. Pathog..

[11] Filardo S., Di Pietro M., Porpora M.G., Recine N., Farcomeni A., Latino M.A., Sessa R. (2017). Diversity of Cervical Microbiota in Asymptomatic *Chlamydia trachomatis* Genital Infection: A Pilot Study. Front. Cell Infect. Microbiol..

[12] Elwell C., Mirrashidi K., Engel J. (2016). Chlamydia cell biology and pathogenesis. Nat. Rev. Microbiol..

[13] Reddick L.E., Alto N.M. (2014). Bacteria fighting back: How pathogens target and subvert the host innate immune system. Mol. Cell..

[14] Boxx G.M., Cheng G. (2016). The Roles of Type I Interferon in Bacterial Infection. Cell. Host. Microbe..

[15] Barker J.R., Koestler B.J., Carpenter V.K., Burdette D.L., Waters C.M., Vance R.E., Valdivia R.H. (2013). STING-Dependent Recognition of Cyclic di-AMP Mediates Type I Interferon Responses during *Chlamydia trachomatis* Infection. mBio.

[16] McNab F., Mayer-Barber K., Sher A., Wack A., O’Garra A. (2015). Type I interferons in infectious disease. Nat. Rev. Immunol..

[17] Rottenberg M.E., Gigliotti Rothfuchs A.C., Gigliotti D., Svanholm C., Bandholtz L., Wigzell H. (1999). Role of innate and adaptive immunity in the outcome of primary infection with *Chlamydia pneumoniae*, as analyzed in genetically modified mice. J. Immunol..

[18] Cotter T.W., Ramsey K.H., Miranpuri G.S., Poulsen C.E., Byrne G.I. (1997). Dissemination of *Chlamydia trachomatis* chronic genital tract infection in gamma interferon gene knockout mice. Infect. Immun..

[19] Johansson M., Schön K., Ward M., Lycke N. (1997). Genital tract infection with *Chlamydia trachomatis* fails to induce protective immunity in gamma interferon receptor-deficient mice despite a strong local immunoglobulin A response. Infect. Immun..

[20] Perry L.L., Feilzer K., Caldwell H.D. (1997). Immunity to *Chlamydia trachomatis* is mediated by T helper 1 cells through IFN-gamma-dependent and -independent pathways. J. Immunol..

[21] Filardo S., Di Pietro M., Tranquilli G., Latino M.A., Recine N., Porpora M.G., Sessa R. (2019). Selected Immunological Mediators and Cervical Microbial Signatures in Women with *Chlamydia trachomatis* Infection. mSystems.

[22] Filardo S., Skilton R.J., O’Neill C.E., Di Pietro M., Sessa R., Clarke I.N. (2019). Growth kinetics of *Chlamydia trachomatis* in primary human Sertoli cells. Sci. Rep..

[23] Sessa R., Di Pietro M., Filardo S., Bressan A., Mastromarino P., Biasucci A.V., Rosa L., Cutone A., Berlutti F., Paesano R. (2017). Lactobacilli-lactoferrin interplay in *Chlamydia trachomatis* infection. Pathog. Dis..

[24] Sessa R., Di Pietro M., De Santis F., Filardo S., Ragno R., Angiolella L. (2015). Effects of Mentha suaveolens essential oil on *Chlamydia trachomatis*. Biomed. Res. Int..

[25] Mastromarino P., Di Pietro M., Schiavoni G., Nardis C., Gentile M., Sessa R. (2014). Effects of vaginal lactobacilli in *Chlamydia trachomatis* infection. Int. J. Med. Microbiol..

[26] Newman L., Rowley J., Vander Hoorn S., Wijesooriya N.S., Unemo M., Low N., Stevens G., Gottlieb S., Kiarie J., Temmerman M. (2015). Global Estimates of the Prevalence and Incidence of Four Curable Sexually Transmitted Infections in 2012 Based on Systematic Review and Global Reporting. PLoS ONE.

[27] Billiau A., Matthys P. (2009). Interferon-gamma: A historical perspective. Cytokine. Growth. Factor. Rev..

[28] Chiliveru S., Birkelund S., Paludan S.R. (2010). Induction of interferon-stimulated genes by *Chlamydia pneumoniae* in fibroblasts is mediated by intracellular nucleotide-sensing receptors. PLoS ONE.

[29] Joyee A.G., Yang X. (2008). Role of toll-like receptors in immune responses to chlamydial infections. Curr. Pharm. Des..

[30] Lad S.P., Fukuda E.Y., Li J., de la Maza L.M., Li E. (2005). Up-regulation of the JAK/STAT1 signal pathway during *Chlamydia trachomatis* infection. J. Immunol..

[31] Di Pietro M., Filardo S., Alfano V., Pelloni M., Po A., Paoli D., Ferretti E., Sessa R. (2019). *Chlamydia trachomatis* elicits TLR3 expression but disrupts the inflammatory signaling down-modulating NFκb and IRF3 transcription factors in human Sertoli cells. Front. Microbiol..

[32] Beckett E.L., Phipps S., Starkey M.R., Horvat J.C., Beagley K.W., Foster P.S., Hansbro P.M. (2012). TLR2, but not TLR4, is required for effective host defence against Chlamydia respiratory tract infection in early life. PLoS ONE.

[33] Derbigny W.A., Shobe L.R., Kamran J.C., Toomey K.S., Ofner S. (2012). Identifying a role for Toll-like receptor 3 in the innate immune response to *Chlamydia muridarum* infection in murine oviduct epithelial cells. Infect. Immun..

[34] Prantner D., Darville T., Nagarajan U.M. (2010). Stimulator of IFN gene is critical for induction of IFN-beta during *Chlamydia muridarum* infection. J. Immunol..

[35] Al-Zeer M.A., Al-Younes H.M., Lauster D., Abu Lubad M., Meyer T.F. (2013). Autophagy restricts *Chlamydia trachomatis* growth in human macrophages via IFNG-inducible guanylate binding proteins. Autophagy.

[36] Rothfuchs A.G., Gigliotti D., Palmblad K., Andersson U., Wigzell H., Rottenberg M.E. (2001). IFN-alpha beta-dependent, IFN-gamma secretion by bone marrow-derived macrophages controls an intracellular bacterial infection. J. Immunol..

[37] Entrican G., Wattegedera S., Rocchi M., Fleming D.C., Kelly R.W., Wathne G., Magdalenic V., Howie S.E. (2004). Induction of inflammatory host immune responses by organisms belonging to the genera *Chlamydia/Chlamydophila*. Vet. Immunol. Immunopathol..

[38] Imtiaz M.T., Schripsema J.H., Sigar I.M., Ramsey K.H. (2006). Outcome of urogenital infection with *Chlamydia muridarum* in CD14 gene knockout mice. BMC Infect. Dis..

[39] Yang C., Briones M., Chiou J., Lei L., Patton M.J., Ma L., McClarty G., Caldwell H.D. (2019). *Chlamydia trachomatis* Lipopolysaccharide Evades the Canonical and Noncanonical Inflammatory Pathways To Subvert Innate Immunity. mBio.

[40] Shima K., Kaeding N., Ogunsulire I.M., Kaufhold I., Klinger M., Rupp J. (2018). Interferon-γ interferes with host cell metabolism during intracellular *Chlamydia trachomatis* infection. Cytokine.

[41] Oliveira-Nascimento L., Massari P., Wetzler L.M. (2012). The Role of TLR2 in Infection and Immunity. Front. Immunol..

[42] John S.P., Sun J., Carlson R.J., Cao B., Bradfield C.J., Song J., Smelkinson M., Fraser I.D.C. (2018). IFIT1 Exerts Opposing Regulatory Effects on the Inflammatory and Interferon Gene Programs in LPS-Activated Human Macrophages. Cell. Rep..

[43] Tietzel I., El-Haibi C., Carabeo R.A. (2009). Human guanylate binding proteins potentiate the anti-chlamydia effects of interferon-gamma. PLoS ONE.

[44] Shtrichman R., Samuel C.E. (2001). The role of gamma interferon in antimicrobial immunity. Curr. Opin. Microbiol..

[45] de la Maza L.M., Peterson E.M., Goebel J.M., Fennie C.W., Czarniecki C.W. (1985). Interferon-induced inhibition of *Chlamydia trachomatis*: Dissociation from antiviral and antiproliferative effects. Infect. Immun..

[46] Theofilopoulos A.N., Baccala R., Beutler B., Kono D.H. (2005). Type I interferons (alpha/beta) in immunity and autoimmunity. Annu. Rev. Immunol..

[47] Farrar M.A., Schreiber R.D. (1993). The molecular cell biology of interferon-gamma and its receptor. Annu. Rev. Immunol..

[48] Chen C., Guo L., Shi M., Hu M., Hu M., Yu M., Wang T., Song L., Shen B., Qian L. (2012). Modulation of IFN-γ receptor 1 expression by AP-2α influences IFN-γ sensitivity of cancer cells. Am. J. Pathol..

[49] Green D.S., Young H.A., Valencia J.C. (2017). Current prospects of type II interferon γ signaling and autoimmunity. J. Biol. Chem..

[50] Schreiber G. (2017). The molecular basis for differential type I interferon signaling. J. Biol. Chem..

[51] Ivashkiv B.L., Donlin T.L. (2014). Regulation of type I interferon responses. Nat. Rev. Immunol..

[52] Masson L., Mlisana K., Little F., Werner L., Mkhize N.N., Ronacher K., Gamieldien H., Williamson C., Mckinnon L.R., Walzl G. (2014). Defining genital tract cytokine signatures of sexually transmitted infections and bacterial vaginosis in women at high risk of HIV infection: A cross-sectional study. Sex. Transm. Infect..

[53] Agrawal T., Vats V., Wallace P.K., Salhan S., Mittal A. (2007). Cervical cytokine responses in women with primary or recurrent chlamydial infection. J. Interferon Cytokine Res..

