# Supplementary figures and images for: Interferon-γ Possesses Anti-Microbial and Immunomodulatory Activity on a Chlamydia trachomatis Infection Model of Primary Human Synovial Fibroblasts
Source: Microorganisms. 2020 Feb 10;8(2):235. doi: 10.3390/microorganisms8020235 (PMC7074713; doi:10.3390/microorganisms8020235)

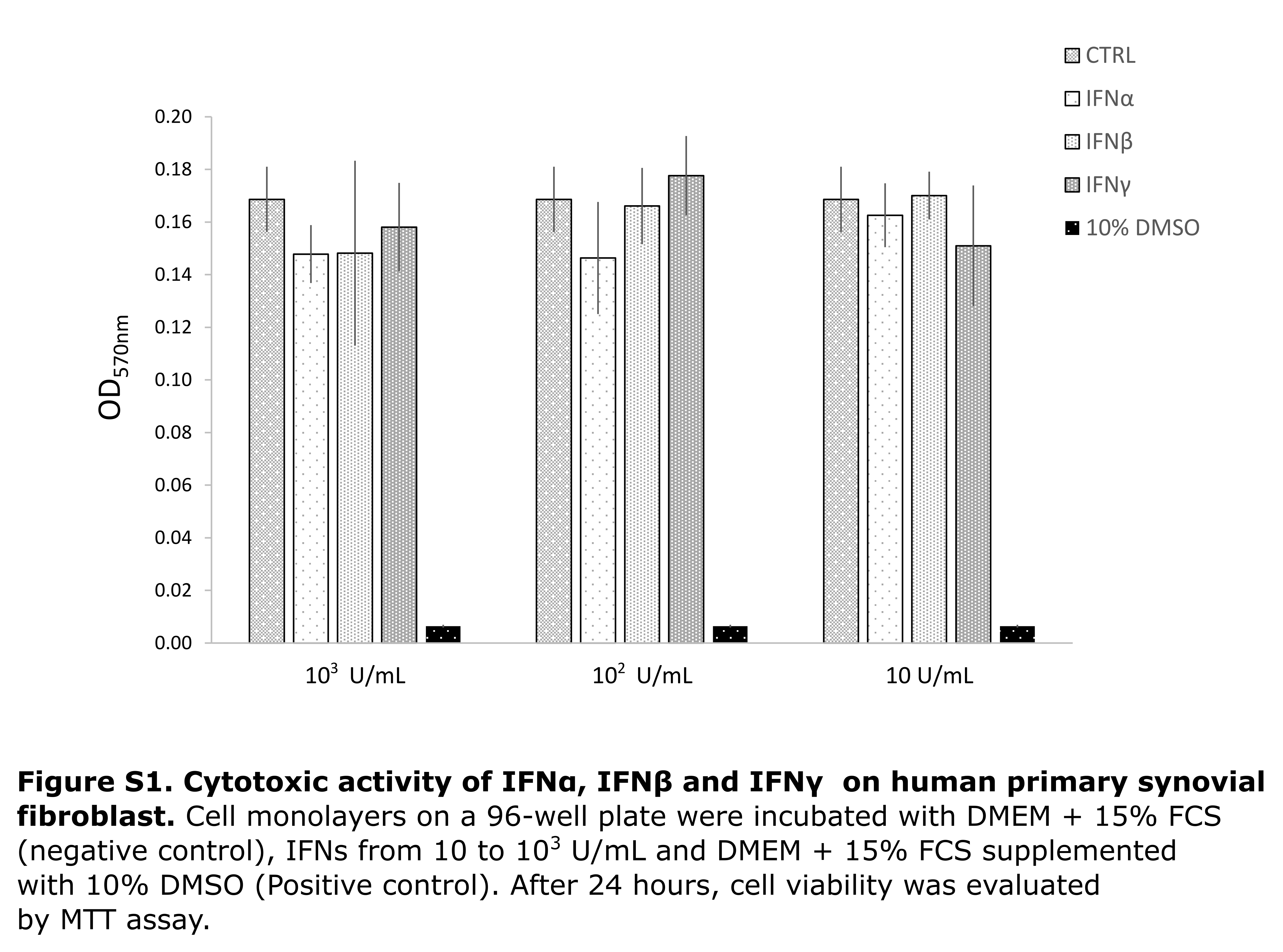

Supplement: Supplementary file 1 [file microorganisms-08-00235-s001.zip › microorganisms-710350-sup.tiff]
